# Cryptic Prophages Contribution for *Campylobacter jejuni* and *Campylobacter coli* Introgression

**DOI:** 10.3390/microorganisms10030516

**Published:** 2022-02-26

**Authors:** Luís Tanoeiro, Mónica Oleastro, Alexandra Nunes, Andreia T. Marques, Sílvia Vaz Duarte, João Paulo Gomes, António Pedro Alves Matos, Jorge M. B. Vítor, Filipa F. Vale

**Affiliations:** 1 Pathogen Genome Bioinformatics and Computational Biology, Research Institute for Medicines (iMed-ULisboa), Faculty of Pharmacy, Universidade de Lisboa, 1649-003 Lisboa, Portugal; luistanoeiro@gmail.com (L.T.); andreia.f.marques@sapo.pt (A.T.M.); jvitor@ff.ulisboa.pt (J.M.B.V.); 2 National Reference Laboratory for Gastrointestinal Infections, Department of Infectious Diseases, National Institute of Health Dr. Ricardo Jorge, 1600-609 Lisboa, Portugal; monica.oleastro@insa.min-saude.pt; 3 Bioinformatics Unit, Department of Infectious Diseases, National Institute of Health Dr. Ricardo Jorge, 1600-609 Lisboa, Portugal; alexandra.nunes@insa.min-saude.pt (A.N.); j.paulo.gomes@insa.min-saude.pt (J.P.G.); 4 Innovation and Technology Unit, Department of Human Genetics, National Institute of Health Dr. Ricardo Jorge, 1600-609 Lisboa, Portugal; silvia.duarte@insa.min-saude.pt; 5 Centro de Investigação Interdisciplinar Egas Moniz (CiiEM), Cooperativa de Ensino Superior Egas Moniz, Quinta da Granja, 2829-511 Caparica, Portugal; apamatos@gmail.com

**Keywords:** bacteriophage, introgression, *Campylobacter*, host range

## Abstract

*Campylobacter coli* and *C. jejuni*, the causing agents of campylobacteriosis, are described to be undergoing introgression events, i.e., the transference of genetic material between different species, with some isolates sharing almost a quarter of its genome. The participation of phages in introgression events and consequent impact on host ecology and evolution remain elusive. Three distinct prophages, named *C. jejuni* integrated elements 1, 2, and 4 (CJIE1, CJIE2, and CJIE4), are described in *C. jejuni*. Here, we identified two unreported prophages, *Campylobacter coli* integrated elements 1 and 2 (CCIE1 and CCIE2 prophages), which are *C. coli* homologues of CJIE1 and CJIE2, respectively. No induction was achieved for both prophages. Conversely, induction assays on CJIE1 and CJIE2 point towards the inducibility of these prophages. CCIE2-, CJIE1-, and CJIE4-like prophages were identified in a *Campylobacter* spp. population of 840 genomes, and phylogenetic analysis revealed clustering in three major groups: CJIE1-CCIE1, CJIE2-CCIE2, and CJIE4, clearly segregating prophages from *C. jejuni* and *C. coli*, but not from human- and nonhuman-derived isolates, corroborating the flowing between animals and humans in the agricultural context. Punctual bacteriophage host-jumps were observed in the context of *C. jejuni* and *C. coli*, and although random chance cannot be fully discarded, these observations seem to implicate prophages in evolutionary introgression events that are modulating the hybridization of *C. jejuni* and *C. coli* species.

## 1. Introduction

*Campylobacter* species related to campylobacteriosis cases shows that 83.9% of the identified bacteria were *C. jejuni*, 10.3% were *C. coli*, while the remaining are other *Campylobacter* species [1]. Thus, the thermotolerant *C. jejuni* and *C. coli* are the major sources of human campylobacteriosis [2,3,4], mostly attributed to poultry meat handling and consumption [5,6]. These two species are thought to have diverged over 6500 years ago during the Neolithic revolution, coinciding with animal domestication and changes in agriculture practices, while *C. coli* population diverged into three distinct clades (clades 1, 2, and 3) by about 1700–1000 years ago [4,7]. The appearance of clonal complexes in the population occurred way after the species divergence [4].

An interesting phenomenon occurring in the natural competent *Campylobacter* spp. [8] is the transference of genetic material between different species, the so-called introgression. *C. jejuni* and *C. coli* from clade 1, which are ca. 12% divergent at nucleotide sequence level (as much as humans are from marmoset) [4], frequently exchange genetic material through horizontal gene transfer. The extensive introgression, more common in *C. jejuni*-to-*C. coli* direction, led to the replacement of ≈10% and ≈23% of *C. coli* core genome with *C. jejuni* DNA, in ST-828 and ST-1150 clonal complexes, respectively. These two clonal complexes in *C. coli* clade 1 arose by *C. jejuni* DNA accumulation and constitute the vast majority of typed isolates from this clade [9]. The introgression levels in the opposite direction or involving *C. coli* isolates from clades 2 and 3 are far less common [9] in the case of the last two, probably due to ecological barriers [4]. Thus, these exchanges may arrive from recent changes in the ecologic niche drew by human activity [10]. Introgression represents a source of adaptive alleles being driven by niche overlap between recipient and donor species, which may result in hybrid speciation [11] contributing to the origin of a new species, or even despeciation [10] resulting in the fusion of two species.

The first prophages within *Campylobacter* genome were described years after the sequencing of *C. jejuni* NCTC 11,168 [12], which harbors no prophages. Three distinct *C. jejuni* integrated elements (CJIE) identified in *C. jejuni* strain RM1221 assume major importance: CJIE1, CJIE2, and CJIE4. CJIE1 is a *Campylobacter* Mu-like phage (also known as CMLP1) [12], apparently inducible with mitomycin C, that encodes several proteins with similarities to bacteriophage Mu and other Mu-like prophages. CJIE2 and CJIE4 are similar prophages and encode few structural proteins. Integrative element CJIE3 has been described but pointed out as an integrative plasmid [12]. Recently, a new integrated element called CJIE5 prophage has been proposed [13]. Infectious CJIE1 and CJIE4 phage particles have been described to be difficult to obtain [14,15,16], supporting CJIE-like prophages to be incomplete or remnant prophages. However, CJIE prophages have been reported to have impact on *Campylobacter* spp. features. Indeed, CJIE1-carrying isolates showed significantly increased adherence and invasion when compared to noncarriers, in a mobility- and growth-independent manner [17]; prophage-encoded DNases were reported to inhibit natural transformation in *C. jejuni* isolates carrying CJIE1, CJIE2, and CJIE4 prophages [18,19]; and it was also shown that the carriage of CJIE1 prophage affects protein expression, including virulence-associated proteins [16].

Whether *C. coli* genomes present prophage homologues of *C. jejuni* prophages or if *Campylobacter* prophages have the ability to infect both *C. jejuni* and *C. coli* remains to be determined and are the aims of the present study. To address these points, we have analyzed 177 *Campylobacter* prophage sequences among 840 genomes, of which 22 *C. jejuni* and 82 *C. coli are* newly sequenced genomes isolated from human and nonhuman hosts, and 692 *C. jejuni* and 44 *C. coli* are genomes available in public databases.

## 2. Materials and Methods

### 2.1. Campylobacter Genomes

A total of 104 genomes from nonrelated Portuguese *Campylobacter* spp. isolates, 22 *C. jejuni* (15 clinical and 7 poultry) and 82 *C. coli* (43 clinical and 39 poultry) (Table S1) were selected for prophage screening. Whole genome sequencing (WGS) and de novo assembly were performed as previously described [20]. Raw sequence reads of six strains (four *C. coli* and two *C. jejuni*) representative of the prophages diversity were deposited in the European Nucleotide Archive (ENA) under the study accession numbers PRJEB46733 and PRJEB46750, respectively. For comparative purposes, prophage screening was also evaluated on 692 *C. jejuni* and 44 *C. coli* genomes retrieved from PATRIC [20].

### 2.2. Prophage Screening Using Bioinformatic Tools

PHASTER [21] and Prophage Hunter Tool (PHT) [22] were used for identification of potential prophagic regions in the newly sequenced *C. coli* and *C. jejuni* genomes, considering only regions predicted as intact (by PHASTER) or active (by PHT). An additional 25,000 bp minimum region length was established, as over 90% (66/72) of *Campylobacter* spp. phage genomes available at PATRIC are >25,000 bp. The insertion sites were determined using the reference genome *C. coli* 15-537,560 (GenBank Accession: CP006702) [23] as prophageless template. Prophages were annotated using RAST [24], and further sequence analysis was done using BLAST [25], while structural homology analyses were performed using Phyre2 [26].

### 2.3. Prophage Induction

Prophage induction was performed using 2 µg/mL mitomycin C [27,28] or 0.15% sodium deoxycholate [15], as described elsewhere [15,27,28] for the *C. jejuni* strains Cj7 and Cj18 (harboring CJIE1- (MZ667637) and CJIE2-like (MZ667636) prophages, respectively), and *C. coli* strains Cc84 and Cc11 (harboring CCIE1- (MZ667638) and CCIE2-like (MZ667639) newly identified prophages, respectively). Phage-induced lysis was tested in bacterial lawns of potentially indicator strains *C. jejuni* Cj11 and *C. coli* Cc88 (predicted to harbor no prophage).

Phage DNA from concentrated phage particles was extracted with QIAprep Spin Miniprep Kit (Qiagen, Germantown, MD, USA), following manufacturer’s instructions for large plasmid (>10 kb). To ensure complete bacterial DNA elimination, sequential digestion with Exonuclease I (*E. coli*) (New England Biolabs, Ipswich, MA, USA) and Lambda Exonuclease (New England Biolabs) was performed as described elsewhere [29]. Presence of phagic and bacterial DNA was tested by PCR targeting CJIE1-CCIE1 morphogenesis protein gene, CJIE2-CCIE2 terminase gene, and *Campylobacter* spp. glutamine synthetase gene (Table S2). Phage particles were observed by transmission electron microscopy (JEOL 100SX) after negative staining, as previously described [29].

### 2.4. Phylogenetic Analysis

After MAFFT version 7 [30] alignment, a maximum-likelihood phylogenetic tree using Jukes–Cantor model of nucleotide evolution [31] was constructed with FastTree 2.1 [32]. Prophages CJIE1-1 to 1-4 (HM141978, HM192820, HM581889, and HM543163) and CJIE4-1 to 4-5 (KF751793, KF751794, KF751795, KF751796, and KF751797), as well as CCIE2 prophage (MZ667634), were included as model prophages representative of each subtree. The CJIE2 and CJIE3 regions of *C. jejuni* RM1221 (NC_003912.7) were extracted and included. Enterobacteria phage Mu (NC_000929) [33] was included as outgroup. Trees were visualized using Interactive Tree Of Life (iTOL) v4 [34].

### 2.5. Testing Introgression Using ABBA-BABA Statistics

ABBA-BABA statistics, or D statistics, was used to test for introgression using single nucleotide polymorphism (SNP) data [35,36,37,38], allowing to determine if introgression has occurred, and between which taxa, based on expectations for the frequencies of different gene tree topologies [39]. An excess of a SNP pattern is indicative of introgression, i.e., gene flow between two of the taxa [37]. The introgression was tested for CJIE4 and for CCIE2 and CJIE2 prophages, since these were the cases where prophage spillover between *C. jejuni* and *C. coli* species was detected by phylogenetic analysis. A multiple sequence alignment using MAFFT [30] was performed for the CJIE4 prophages and another for CCIE2 and CJIE2 prophages, using in both cases Enterobacteria phage Mu (NC_000929) [33] as outgroup. SNPs were extracted from multiple sequence alignments using SNP-sites [40]. Using an R script, the allele frequencies at each SNP were determined, followed by D statistic and block jackknife method to test for a significant deviation from the null hypothesis D = 0 [35,36]. The admixture proportion was determined using fd statistic [37,38,41].

### 2.6. Prophage Nuclease Screening

The identified prophages were screened for nucleases using CJIE1-encoded endonuclease *dns* (*locus* tag: CJE0256 in *C. jejuni* RM1221), or the CJIE2- and CJIE4-encoded endonuclease *nucA* (*locus* tag: CJE0566 in *C. jejuni* RM1221 and *locus* tag: 01-1512_00025 in *Campylobacter* phage CJIE4-5, respectively) [18,19]. In either case, genes with coverage >90% were considered as complete genes, while genes with coverage 50–90% were considered as partial. Lower coverages were reported as not detected.

## 3. Results

### 3.1. Identification of Prophages

Within the 22 *C. jejuni* and 82 *C. coli* newly sequenced genomes, a total of 402 prophage regions were predicted (123 PHASTER-identified and 279 PHT-identified, mean length of 18,340.47 ± 9773.85 bp, ranging from 4543 to 50,845 bp—data not shown). Considering only predicted prophages without homology with plasmids, larger than 25,000 bp, presenting structural proteins, and classified as intact (by PHASTER) or active (by PHT) reduces the list to nine by PHASTER (2 in *C. jejuni* and 7 in *C. coli*) and 29 by PHT (4 in *C. jejuni* and 25 in *C. coli*) (Table 1). PHASTER and PHT clearly identified three groups of prophages with homology with CJIE1, CJIE2, and CJIE4. The nine PHASTER-identified prophages evidenced homology with CJIE1 (average percent identity of 81.1% for *C. coli* and 93.1% for *C. jejuni* predicted prophages). Among the PHT-predicted prophages, 28 had homology with CJIE2 (average percent identity of 54.5% for *C. coli* and 52.8% for *C. jejuni* predicted prophages), and one with CJIE4 (percent identity of 91.7% for a *C. coli* prophage). These observations together with the phylogenetic analysis (see below) pinpoint the existence of two novel prophages in *C. coli* that for their similarity with *C. jejuni* prophages were named *Campylobacter coli* integrated element 1 (CCIE1) and *Campylobacter coli* integrated element 2 (CCIE2). Although CJIE1-like prophages have been considerably reported in *C. coli* genomes [42], the majority (if not all) of them may be, in fact, CCIE1 prophages. Interestingly, CJIE2 prophages were only reported in *C. jejuni* isolates so far [12,42,43,44,45,46,47], whereas, in contrast, CCIE2 prophages were found in both *C. coli* and *C. jejuni* genomes. CCIE1 and CJIE1 are very similar to each other (≈80% sequence identity), while CCIE2 and CJIE2 do not show such similarity (≈50% sequence identity). Significant deletions and rearrangements in CJIE2-like prophages were described [44,45], potentially explaining the low coverage of homologous regions when comparing CJIE2 and CCIE2 prophages. Notably, CJIE1-CCIE1 and CJIE2-CCIE2 display several genes shared in block with same organization (data not shown). Both CCIE1 and CCIE2 showed genome length of 38,556 bp and 36,356 bp, respectively, consistent with CJIE1 (34,403 bp) and CJIE2 (40,268 bp).

### 3.2. Characterization of the New CCIE1 and CCIE2 Prophages

**Figure 1:**
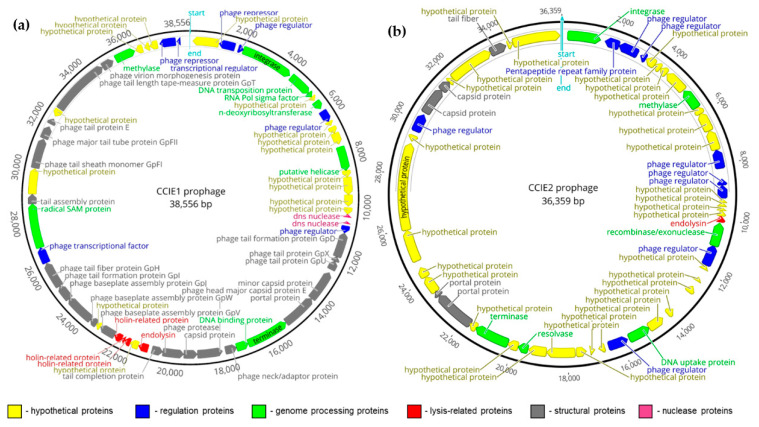
Genome annotation of the newly identified CCIE1 (**a**) and CCIE2 (**b**) prophages. The genome of both CCIE1 and CCIE2 prophages and their annotated CDS are represented. Hypothetical proteins for which no annotation update was possible are represented in yellow. Regulation proteins and genome processing proteins are depicted, respectively, in blue and green, while lysis-related proteins and structural proteins are highlighted in red and grey, respectively. Partial dns nuclease found in CCIE1 is shown in lilac. For space simplification, the linear prophage region is represented as a circular genome, with start (first residue) and end (last residue) highlighted at 0′ position of each representation. The figure was obtained using Geneious Prime 2020.1.1. Further description of the annotated regions is available on Table S3.

A more detailed analysis was performed on the CCIE1 region within *C. coli* Cc63-H-18 genome, and on the CCIE2 region within *C. coli* Cco1598-H-13 genome (Figure 1, Table 2 and Table S3). Most predicted CDS in both CCIE1 e CCIE2 were matched either by sequence or structure with phage genes (Table S3). Several hypothetical proteins were annotated as a result of the lack of knowledge surrounding *Campylobacter* spp. phages. A bacterial putative NADH-ubiquinone oxidoreductase, located at the 3′ edge of CCIE2 genome, was identified, potentially representing a watermark from a past phage transduction event. More specifically, the peripheral location of the putative bacterial gene in the prophage genome points to specialized transduction, in which the bacteriophage packages its genome with flanking bacterial DNA taken during chromosomal excision [48].

Regarding structural analysis, CCIE1 seems a near-complete prophage lacking only one essential structural protein, possibly explaining the general failure of induction attempts of its close homologue CJIE1 prophage [15,49], while not objecting a case of apparent CJIE1 induction success [12]. However, it should not be ruled out that induction failure may be due to inefficient experimental conditions. On the other hand, CCIE2 was predicted to have several structural proteins missing, similar to its homologue CJIE2 [12]. The lack of structural proteins is a common feature of cryptic prophages [50], which suggests that CCIE2 is possibly incomplete.

**Table 2:** General characterization of the newly identified CCIE1 and CCIE2 prophages.

Characteristics	CCIE1 Prophage		CCIE2 Prophage
Genome length		38,556 bp	36,356 bp
GC content		30.20%	28.50%
GenBank Accession No.		MZ667635	MZ667634
Closest homologue		CJIE1-2 (HM192820.1) [51]	CJIE2 [12]
Coverage with the closest homologue		79%	54%
Identity with the closest homologue		96.33%	93.85%
Host strain ^1^		*C. coli* Cc63-H-18	*C. coli* Cco1598-H-13
Insertion site ^2^	5′	Bis-ABC ATPase YbiT (N149_0417)	tRNA-Leu-GAG (N149_0910)
	3′	putative lipoprotein (N149_01930)	putative NTPase (N149_01865)
Number of CDS ^3^		59	54
Main phage genes detected ^3^		Integrase, dns nuclease, endolysin, holin, methylase, terminase, several phage structural and regulation proteins.	Integrase, endolysin, recombinase/exonuclease, methylase, resolvase, terminase, several phage structural and regulation proteins.
VIRFAM analysis		Almost complete Mu-like Myoviridae phage (head closure protein missing)	Incomplete phage (several structural proteins missing)

^1^ Raw sequence reads were deposited in the European Nucleotide Archive (ENA) under the study accession number PRJEB46733; ^2^ *locus* tag in the genome of the reference strain *C. coli* 15-537560; ^3^ further details on Table S3.

### 3.3. Prophage Induction Assays

**Figure 2:**
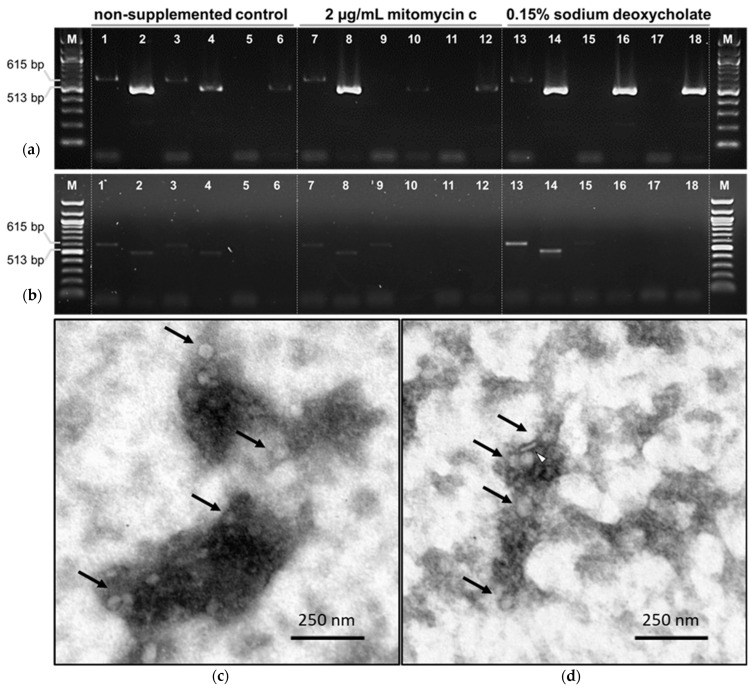
Prophage induction assays. (**a**,**b**) PCR detection of CJIE1 (**a**) and CCIE1 (**b**) circular phagic DNA targeting CJIE1-CCIE1 phage morphogenesis protein gene (513 bp amplicon) and the bacterial glutamine synthetase gene (615 bp amplicon). Odd lanes refer to bacterial gene amplification and even lanes refer to phage gene amplification. The first pair of each induction condition refer to untreated DNA, while the second pair refer to Exonuclease I only and the third to Exonuclease I and Lambda Exonuclease double-treated DNA. Results are shown for non-supplemented control (lanes 1–6), mitomycin C induction (lanes 7–12), and sodium deoxycholate induction (lanes 13–18). M, 100 bp DNA Ladder (NEB). (**a**) PCR reactions on DNA extracted from concentrated putative phage particles obtained upon induction of the CJIE1-like prophage. The amplification of the phagic gene following linear DNA elimination by exonuclease treatment suggests the induction of this prophage, even in the non-supplemented control. Similar results were obtained for the inductions of the CJIE2-like prophage (data not shown). (**b**) PCR reactions on DNA extracted from concentrated putative phage particles obtained upon induction of the CCIE1-like prophage. The lack of amplification of the phagic gene following linear DNA elimination by exonuclease treatment suggests that no induction occurred for this prophage. Similar results were obtained for the inductions of the CCIE2-like prophage (data not shown). (**c**,**d**)—Negative staining transmission electron microscopy images obtained after induction of CJIE1 (**c**) and CJIE2 (**d**) prophages. Roughly icosahedral phage-like particles were observed (arrows) with a diameter of 49 ± 4 nm (CJIE1, (**c**)) and 50 ± 3 nm (CJIE2, (**d**)). Although several tail-related proteins were annotated in the genome, no tail-like structures were observed on CJIE1 particles (**c**), and tail-resembling structures were inconsistently observed for CJIE2 (white arrow head on (**d**)).

Despite being reported as mitomycin C-inducible [12], to our knowledge CJIE1 induction was not described in detail and there are a couple reports of induction failure [15,16]. CJIE2, on the other hand, was reported as incomplete; thus, likely not inducible [12]. For the CJIE1-like prophage, PCR after exonuclease treatments rendered phagic gene amplification (Figure 2a, lanes 6, 12, and 18). Similar results were obtained for the CJIE2-like prophage (data not shown), suggesting that both *C. jejuni*-harbored prophages are inducible. The detection of circular phagic genomes of not-induced bacteria (noninduced control), points towards a basal spontaneous release of phage particles. For the CCIE1-like prophage, PCR after exonuclease treatments rendered no phagic gene amplification (Figure 2b, lanes 6, 12, and 18). Similar results were obtained for the CCIE2-like prophage (data not shown), suggesting that CCIE1 and CCIE2 were not inducible, thus rendering no PCR amplification. Despite these observations, no phage-induced lysis was observed upon application of PCR positive phage precipitates on the potentially indicator strains (data not shown).

Negative staining transmission electron microscopy was performed on PCR-positive phage precipitates, and phage-like structures were observed (arrows on Figure 2c,d). Although scarce, such structures were observed in all the PCR-positive phage precipitates, showing roughly icosahedral heads with 49 ± 4 nm (CJIE1—Figure 2c) and 50 ± 3 nm (CJIE2—Figure 2d). Even though several tail-related proteins were annotated in the genome and predicted by VIRFAM, no tail-like structures were observed on CJIE1 particles (Figure 2c) and tail-resembling structures were inconsistently observed for CJIE2 (Figure 2d, white arrow head). This feature may be due to the lack of tail-related proteins rendering tailless phages or possibly due to some limitations on negative staining which may not reveal phage tails because of their limited density [28]. The observed bacteriophage-like structures conjugated with the PCR detection of circular phage DNA on culture supernatants upon induction points to the production of CJIE1 and CJIE2 bacteriophage particles, thus not supporting the previous reports of CJIE1 and CJIE2 as incomplete, at least in the assayed *Campylobacter* spp. strains.

### 3.4. Identification of CJIE1-, CJIE4-, and CCIE2-like Prophages within Campylobacter spp.

**Figure 3:**
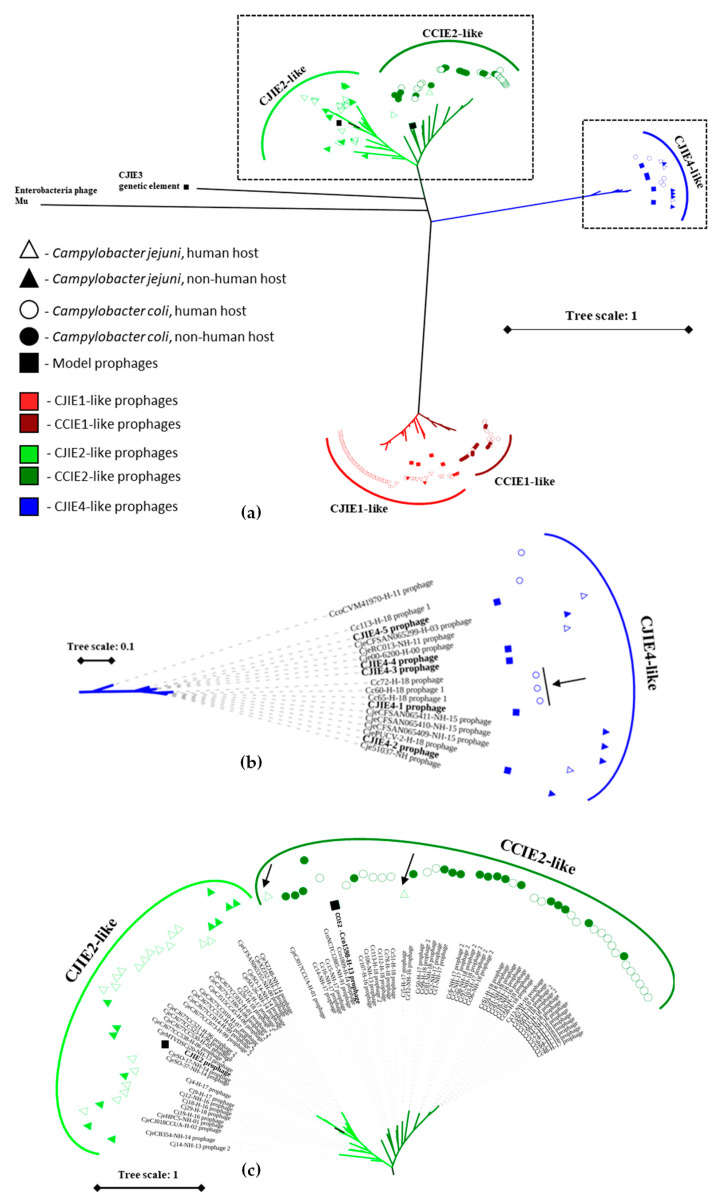
Phylogenetic tree of the identified CJIE1-like, CCIE2-like, and CJIE4-like prophages (**a**) and potential prophages host-jumps (**b**,**c**). CCIE2-like prophages are represented in green, CJIE1-like prophages are represented in red, and CJIE4-like prophages are represented in blue. The model prophages CJIE1-1 to 1-4, CJIE4-1 to 4-5, and CCIE2 prophage are represented as squares. CJIE2 and CJIE3 regions were extracted from *C. jejuni* RM1221 genome, included and represented as squares. Enterobacteria phage Mu is included as an outgroup and used to root the final tree. Prophages harbored by *C. coli* are represented as circles while prophages harbored by *C. jejuni* are represented as triangles. Filled markers are displayed for *Campylobacter* spp. isolates obtained from nonhuman hosts, while isolates from human hosts display unfilled markers. On CCIE2-like cluster, two internal clusters can be distinguished: one composed of *C. jejuni*-harbored prophages (light green) that includes CJIE2 prophage (square); and the other composed majorly of *C. coli*-harbored prophages (dark green) that include the new identified CCIE2 prophage (square), potentially representing a *C. coli* homologue of CJIE2 not yet described. Similarly, on the CJIE1-like cluster, two internal clusters can be distinguished: one composed of *C. jejuni*-harbored prophages (light red) that includes all CJIE1 series prophages (squares); and the other composed of *C. coli*-harbored prophages (dark red) including CCIE1, a not-yet-described *C. coli* homologue of CJIE2. Clusters depicting potential host jumps are highlighted (dashed rectangles) and are presented in (**b**,**c**). Cluster (**b**) refers to CJIE4-like prophages (blue). Cluster (**c**) refers to CCIE2-like prophages (green). No potential host-jumps were detected in CJIE1-like prophages (red cluster on (**a**)). Potential introgression events are emphasized (arrows). Tree scales and key are presented. Prophages used to construct the tree are detailed on Table S4.

To understand the phage dynamics on *Campylobacter* spp. population, the reference CJIE1-1 and CJIE4-1 prophages, as well as the newly identified CCIE2 prophage, were used as template model prophages, since the prophages identified using PHASTER and PHT fell essentially in these three groups (Figure 3a). Integrated elements CJIE3 and CJIE5 were not included as models for this screening as neither PHASTER- nor PHT-predictions evidenced any of these prophages. To double-check this, BLASTn querying CJIE3 and CJIE5 were conducted, identifying no CJIE3-like regions in the newly sequenced *C. coli* and *C. jejuni* genomes, but verifying the presence of CJIE5 in all genomes. Indeed, CJIE5, which is likely not a prophage, is also present in *C. jejuni* RM1221 (where all were initially identified) and in *C. jejuni* NCTC11168 (described as a prophageless strain). The total number of prophages found was 177 (Table 3 and Table S1). In detail, the screening on the 104 genomes of this study rendered a total of 39 *Campylobacter* spp. isolates carrying CJIE1-like prophages, 53 carrying CCIE2-like prophages, and 4 carrying CJIE4-like prophages. Among these genomes, 12.5% (13/104) harbored two prophages, either CJIE1 and CJIE2 or CJIE2 and CJIE4 (consult Tables S1 and S4 for details). Screening on 736 publicly available genomes rendered a total of 48 *Campylobacter* spp. isolates carrying CJIE1-like prophages, 24 carrying CCIE2-like prophages, and 9 carrying CJIE4-like prophages (Table 3). Among these genomes, 1.1% (8/736) harbored two prophages, CJIE1 and CJIE2 (Table S5). The prophage presence ratio on Portuguese isolates was much higher than in the publicly available genomes of *Campylobacter* spp. (Table 3), and it does not appear to be related with ST or CC type (Tables S1, S4 and S5). Different *Campylobacter* spp. populations, either in terms of location, host of isolation, or phenotypic characteristics, seem to differentially carry CJIE elements (Table S6), supporting a potential role of these prophages in the modulation of carriers genomic and phenotypic features [14,16,17,52], and in the evolution and ecological adaptation of the isolates (as reviewed by Harrison and Brockhurst (2017) [53]). However, our approach of considering only prophages with template coverage over 50% may have led to the discard of prophages, namely remnant, truncated, or mosaic prophages, which may have been analyzed in the previously mentioned studies. Nonetheless, the reverse reasoning is also valid: it is possible that we have considered distinct phages to be similar to the reference phages due to sequence coverage above the 50% mark. For databases-retrieved isolates, we should remind that only isolates with information regarding MLST, country of isolation, and host organism were selected, which may have led to the non-analysis of prophages eventually carried by the remaining genomes available in the databases.

### 3.5. Insertion Sites of the Prophages Identified

The insertion site of CJIE1-like prophages was the least conserved, with over a dozen insertion sites identified (Table S4A). This volatility of insertion sites of CJIE1-CCIE1 prophages is in agreement with other previous descriptions of varied insertion sites [12,42,49,54,55]. Even with diverse insertion sites in the population, CJIE1-like prophages of isolates of the same ST largely shared the same integration sites, especially in the context of *C. coli*. Interestingly, the original insertion site of CJIE1 in *C. jejuni* RM1221 was not between the determined insertion sites [12].

Among CCIE2-like prophages, the insertion sites were highly conserved, pointing to vertical prophage transmission, with prophages within *C. coli* genomes normally inserting between a tRNA-Leu gene (*locus* tag: N149_0910) and an adjacent hypothetical protein coding sequence, identified as a putative NTPase or cell division-related protein (*locus* tag: N149_01865) (Table S4B). *C. jejuni* CCIE2-like prophage genomes were usually inserted between a tRNA-Arg gene and an adjacent hypothetical protein coding sequence, which could not be identified either by sequence or structure homology (*locus* tag: Cj0494) (Table S4B). Consistent with previous observations, this insertion site is the same used in *C. jejuni* RM1221 by CJIE2 [12], which was also described to be majorly conserved [44,45,47].

Similarly, most CJIE4-like prophages shared a common insertion site in *C. jejuni* isolates between a tRNA-Met (upstream of *locus* tag: Cj1282) and a tRNA-Phe genes (downstream of *locus* tag: Cj1283) (Table S4C). This insertion site transversal to *C. jejuni* isolates is coincident with the original insertion site of CJIE4 in *C. jejuni* RM1221 where it was first identified [12], and with previous reports [14,54], reinforcing the conservation of this prophage insertion site. The insertion site for CJIE4-like prophages harbored by *C. coli* isolates was hard to determine precisely, but generally seems to be between a tRNA-Met gene (*locus* tag: N149_0550, described in the reference genome as a tRNA-Ile gene) and an adjacent hypothetical protein-coding sequence, identified as a putative conjugative transfer protein (upstream of *locus* tag: N149_0550) (Table S4C).

### 3.6. Phylogenetic Analysis of the Prophages Identified

The maximum-likelihood phylogenetic tree (Figure 3a) clearly separated the three groups of prophages identified, CJIE1-, CJIE4-, and CCIE2-like prophages. Inside each group, the separation between prophages harbored by human-derived isolates (unfilled markers) and nonhuman-derived isolates (filled markers) was weak, as expected by the typical epidemiology and transmission mechanisms of *Campylobacter* spp. with isolates flowing between animals and humans in the agricultural context suggested by the clonal complexes of isolates [4]. CJIE3 did not cluster with either of these groups, highlighting its genomic divergence. In the CJIE1- and CCIE1-like prophages cluster, there was a clear separation between *C. coli* and *C. jejuni* harbored prophages. The cluster of CJIE4-like prophages (blue markers) was more well mixed, with three prophages harbored by *C. coli* isolates (Cc60-H-18, Cc65-H-18, and Cc72-H-18, arrowed in Figure 3b) being displayed among the *C. jejuni*-carried prophages and not closer to the remaining prophages harbored by the *C. coli* isolates, especially the early-diverging CcoCVM41970-H-11 prophage (Figure 3b). Although the isolate Cc72-H-18 has a new reported MLST profile with no clonal complex assigned, the isolates Cc60-H-18 and Cc65-H-18 belong to ST-828 complex, within *C. coli* clade 1, for which introgression events were described [9]. These three prophages can point to potential host-jumps and introgression events, but a larger set of CJIE4-like prophages would be necessary for strong conclusions. To test introgression, the ABBA-BABA test was performed [35,36], and when introgression was detected, the admixture proportion was determined [37,38]. Comparing the genomes of prophages reveals that CJIE4-like prophage of *C. coli* genomes Cc72, Cc60_1, and Cc65_1 (arrow in Figure 3b) and CJIE4-like prophage from *C. jejuni* genomes share more derived alleles than expected by chance. The resulting positive D-statistic of 0.6426 and Z-score of 7.583 (Table S7A) suggests introgression between these prophages, with an estimated admixture of 88.31% (Table S7A).

For CCIE2-like prophages, a sharp separation between *C. coli*-harbored and *C. jejuni*-harbored prophages was observed (Figure 3c), segregating CJIE2- and CCIE2-like prophages. The CJIE2 prophage integrates the cluster comprising only prophages harbored by *C. jejuni* isolates, while CCIE2 prophage was in the cluster comprising majorly CCIE2-like prophages harbored by *C. coli* isolates. In this last cluster, two noticeable exceptions were found: the CCIE2-like prophages carried by *C. jejuni* Cj5-H-17 and CjeCJ017CCUA-H-01 (arrows on Figure 3c). These punctual observations suggest that some *Campylobacter* spp. bacteriophages could have the ability to infect both *C. coli* and *C. jejuni* isolates in interspecies infections, as both contributor and consequence phenomena of introgression events. The ABBA-BABA test verified the existence of prophage introgression for CCIE2-like prophages in *C. jejuni* prophages. Comparing the genomes of prophages reveals that CCIE2-like prophage of *C. jejuni* genomes CjeCJ017CCUA and Cj5-H-17 (arrow in Figure 3C) and CCIE2-like prophage from *C. coli* genomes share more derived alleles than expected by chance. Again, the resulting positive D-statistic of 0.3696 and Z-score of 4.958 (Table S7B) suggests introgression between these prophages, with an estimated admixture of 39.32% (Table S7B). Additionally, the positive D-statistic of 0.3144 with a Z-score of 5.572 suggests introgression of CJIE2 in CCIE2 present in *C. jejuni* genomes, with an estimated admixture of 39.46% (Table S7B).

### 3.7. Newly Identified MLST Allelic Variations and MLST Profiles

Among the analyzed *C. coli* genomes, two new allelic variations and eight new MLST profiles were identified and submitted to PubMLST database [56]. The new profiles were assigned a new non-existing ST with eight being assigned to pre-existing clonal complexes and three having no clonal complex assigned (Table S1). As expected, the majority of our new-profile isolates was assigned to ST-1150 and ST-828 clonal complexes, which dominate clinical and farm isolates [4].

### 3.8. Nucleases Encoded by Campylobacter Prophages

For both CJIE1 and CJIE4, the endonuclease genes *dns* and *nucA*, respectively, were described to prevent carrier isolates’ natural transformation [18,19]. CJIE2 also carries a copy of *nucA* identical to the CJIE4 one [19]. As these endonucleases can have a major role on shaping *Campylobacter* spp. genome evolution and population structure by potentially modeling introgression and contributing to the maintenance of stable lineages, nucleases carried by the identified prophages were analyzed (Table 4). Interestingly, all CJIE1 and CCIE1-like prophages carried a version of *dns* gene either in a complete form or in a partial form. All partial forms were found in *C. coli*-harbored prophages (CCIE1-like prophages) while only two *C. coli*-harbored prophages carried the integral *dns* gene, contrarily to *C. jejuni* (Table 4), thus possibly facilitating the *C. jejuni-*to-*C. coli* introgression process, as it is suggested to occur [4], rather than the opposite direction.

## 4. Discussion

Prophages are commonly found in *C. jejuni* and *C. coli* genomes, although their prevalence may be unbalanced worldwide, since the prevalence of detected prophages diverge between studies [42,43,54]. A manual curation of prophages predicted using specific software [21,22] led to the identification of three main prophage types found in *C. jejuni* and *C. coli* genomes, the CCJIE1 and CCIE1 group, the CJIE2 and CCIE2 group, and the CJIE4 group. Among these, we have introduced two new prophages, CCIE1 and CCIE2, found in *C. coli* genomes, whose close homologues are the *C. jejuni* prophages CJIE1 and CJIE2, respectively. The new prophage found was CCIE1, a 38,556 bp region, probably incomplete and homologous to CJIE1. The other new prophage, CCIE2, is a 36,356 bp region that, although harboring a wide range of bacteriophage-like proteins, is probably an incomplete prophage, as supported by the inability to induce this prophage. This region has several common features with CJIE2 prophage identified in *C. jejuni* RM1221 and was found to represent a close homologue of CJIE2 prophage in *C. coli*. Although not unequivocally, induction assays and analysis point towards the possibility of CJIE1 and CJIE2 inducibility, even though at poor induction rates. It is possible that both CJIE1-CCIE1 and CJIE2-CCIE2 prophages are currently on the edge of becoming cryptic prophages, with most lacking essential structural proteins leading to failure upon induction assays, while some maintain a minimal manageable set of essential structural proteins, making possible the (poor) induction observed for these prophages, namely, the *C. jejuni*-harbored homologues.

The prophages found in a large collection of *C. jejuni* and *C. coli* genomes clustered into three main groups, as supported by the phylogenetic tree, including CJIE1-CCIE1, CJIE2-CCIE2, and CJIE4. The genetic similarity between the pairs CJIE1-CCIE1 and CJIE2-CCIE2 and the phylogenetic clustering according to species points to prophage acquisition previous to the speciation event of *C. jejuni* and *C. coli*, followed by co-evolution between prophages and their bacterial hosts [29,57,58,59]. Furthermore, when there is conservation of the prophage insertion site, prophage vertical transmission may be in place [60,61].

The global scenario suggests that all the three analyzed prophage groups (CJIE1-CCIE1, CJIE2-CCIE2, and CJIE4) have different homologues running in either *Campylobacter* species, even for CJIE4-like prophages, leaving open that a *C. coli* homologue of CJIE4 may also exist. However, we may not discard the hypothesis that CJIE4-like prophages switch between host species (*C. jejuni* and *C. coli*), not denoting a canonical prophage sequence for each species. CJIE4-like prophage kept the same insertion site in both *C. jejuni* and *C. coli* and shared the same phylogenetic cluster, suggesting cross-species transmissions, in line with what was observed for streptococcal prophages [62]. In these cases, prophages may be evolving independently from the host bacteria, driven by ecological relatedness rather than evolutionary relatedness of the host bacteria [62].

Phages are known to be highly species-specific, infecting only one species and sometimes only some strains of a certain species [63]. Remarkably, we found prophages from *C. coli* in *C. jejuni* genomes (CCIE2-like prophage) and vice versa (CJIE4-like prophage). Cross-species transmission of prophages have also been described for streptococcal prophages [62], suggesting that some prophages are capable of species spillover that may erode species differences over time. Furthermore, conserved endonuclease genes such as *dns* and *nucA*, which inhibit natural transformation capability of *Campylobacter* spp. [18,19], were identified among the analyzed prophages, suggesting that prophage presence may be associated with more stable lineages after prophage acquisition.

Indeed, we have found punctual introgression events for CJIE4-like and CCIE2-like prophages. The introgression events found in *C. coli* and *C. jejuni* agree with previous observations. It should be noted that *Campylobacter* spp. have a very plastic and dynamic genome evidencing transference of genetic material between *C. jejuni* and *C. coli*. Consequently, there is sharing of several molecular mechanisms [4]. In fact, ST-828 and ST-1150 clonal complexes were originated in clade 1 of *C. coli* by the accumulation of *C. jejuni* DNA [9]. Despite that *C. jejuni* Cj5-H-17 and CjeCJ017CCUA-H-01 are not assigned to a clonal complex, the harbored prophages are phylogenetically close to CCIE2 prophages harbored by *C. coli* isolates belonging to ST-828 complex (clade 1). Introgression events are described to be more common in *C. jejuni*-to-*C. coli* direction [9], but for CCIE2-like prophage the spillover appears to occur from *C. coli*-to-*C. jejuni*. For CJIE4-like prophages, introgression appears to occur in the classic *C. jejuni*-to-*C. coli* direction and involving ST-828 complex (*C. coli* Cc60-H-18 and Cc65-H-18 belong to ST-828 complex, while *C. coli* Cc72-H-18 does not fit in any pre-existing clonal complex). Introgression can impact clinical and ecological traits of *Campylobacter* spp. isolates and suggests the maintenance of cell mechanisms between these two species, potentially facilitating bacteriophage host-jumps. Although present in a smaller number of strains, the introgression events detected point to introgression driven by prophages. Conjugative plasmids have also been associated with introgression events [64], supporting interspecies introgression through horizontal gene transfer. This spillover of genes from one species into the gene pool of another species shakes species boundaries, which are semipermeable especially at specific genome regions [65]. The mechanism behind bacteriophage host-jumps and introgression phenomena may be mediated by the capacity of the phage to infect both species, or by uptake of DNA since *Campylobacter* species are naturally competent [66]. The absence of induction evidence suggests that CCIE1 and CCIE2 prophages are cryptoprophages—incomplete and dysfunctional versions of the prophages that were once able to complete lytic cycles but were domesticated by the host and condemned to perpetual lysogeny [67]. Although defective, it was previously shown that cryptophages can contribute to host fitness [67], potentially explaining the degree of conservation observed. This observation is consistent with the reported difficulties for obtaining infectious particles of the CCIE1 *C. jejuni*-harbored homologue, CJIE1 [15,16]. The same was here observed for CCIE2 phage, suggested as incomplete by VIRFAM analyses and by its similarities with the incomplete CJIE2. Although not assayed in this work, mainly due to lack of harboring-strain availability, CJIE4-like prophages were also reported to be difficult to induce, thus possibly being incomplete [14]. Interestingly, and contrary to the aforementioned [12,15,16], our induction assays suggest CJIE1 and CJIE2 as inducible. Bacteriophages may play a role in introgression, even in the absence of infectious particles production. It would also be interesting to understand whether the punctual host-jumps mentioned can represent an opportunistic transduction. In other words, they can represent the carriage of genomes from putatively incomplete prophages within infectious particles of complete bacteriophages co-infecting the same host. This phenomenon is called molecular piracy and was already described in *Caudovirales* bacteriophages, the order of all *Campylobacter* phages described so far [68,69,70]. Prophage introgression by random chance due to natural competence in *Campylobacter* spp. cannot be fully discarded, and additional work is required to better clarify the role of prophages for population structure. Further studying *Campylobacter* spp. prophage genomics and phylogenetics is of major interest and may give insight not only into bacteriophage biology, but also on *Campylobacter* population structure and introgression phenomena.

## Figures and Tables

**Table 1:** Number of predicted prophage regions within newly sequenced *C. coli* and *C. jejuni* genomes using PHASTER [21] and Prophage Hunter [22].

Detected Prophage Sequences *	*C. jejuni* (*n* = 22)	*C. coli* (*n* = 82)	Total (*n* = 104)
Genomes without prophage sequences	1	5	6
PHASTER (total)	39	84	123
PHASTER (intact)	2	7	9
Prophage Hunter Tool (total)	84	195	279
Prophage Hunter Tool (active)	4	25	29

* Parenthesis: total number of phages and number of phages predicted to be complete by PHASTER (software classification intact) and Prophage Hunter (software classification active).

**Table 3:** Presence of *C. coli*- and *C. jejuni*-harbored prophages in the genomes analyzed.

*Campylobacter* spp. Genomes	CJIE1	CCIE2	CJIE4
*C. coli* genomes from present study	35.37% (29/82)	52.44% (43/82)	4.88% (4/82)
*C. coli* genomes from public databases	0.00% (0/44)	2.27% (1/44)	2.27% (1/44)
*C. jejuni* genomes from present study	45.45% (10/22)	45.45% (10/22)	0.00% (0/22)
*C. jejuni* genomes from public databases	6.94% (48/692)	3.32% (23/692)	1.16% (8/692)
Total per model prophage (*n* = 177)	87	77	13

**Table 4:** Predicted nuclease gene presence among the identified prophages. The endonuclease *dns* (CJIE1-CCIE1) and *nucA* genes (CJIE2-CCIE2 and CJIE4) predicted in the identified prophage are presented.

Prophage	Harboring-Species	Nuclease Gene *	
		Complete	Partial
CJIE1-CCIE1-like prophages	*C. coli* (*n* = 29)	2	27
	*C. jejuni* (*n* = 58)	58	n.d.
CJIE2-CCIE2-like prophages	*C. coli* (*n* = 44)	6	n.d.
	*C. jejuni* (*n* = 33)	18	3
CJIE4-like prophages	*C. coli* (*n* = 5)	1	n.d.
	*C. jejuni* (*n* = 8)	3	n.d.

* Genes with coverage >90% were considered as complete, while genes with coverage 50–90% were considered as partial; n.d., lower coverages were reported as not detected.

## Data Availability

Raw sequence reads of bacterial genomes, which are representative of the prophages diversity, are deposited in the European Nucleotide Archive (ENA), under the study accession numbers PRJEB46733 and PRJEB46750. *Campylobacter* prophage sequences are available under the GenBank accession numbers MZ667634-MZ667639.

## Supplementary Materials

The following supporting information can be downloaded at: https://www.mdpi.com/article/10.3390/microorganisms10030516/s1, Table S1: General characterization of the newly sequenced *Campylobacter* spp. isolates analyzed in the present study; Table S2: Primer pairs used for phage circular DNA detection; Table S3: Predicted annotated regions within CCIE1 and CCIE2 prophage genomes; Table S4: General characterization of the prophages identified in the present study within *Campylobacter* spp. population; Table S5: General characterization of database isolates harboring prophages; Table S6: Presence of *C. coli*- and *C. jejuni*-harbored prophages in different studies; and Table S7: ABBA-BABA test considering the following tree sample organization (((P1, P2) P3) P4)).
